# Continuum of Trauma: Fear and Mistrust of Institutions in Communities of Color During the COVID-19 Pandemic

**DOI:** 10.1007/s11013-023-09835-3

**Published:** 2023-09-30

**Authors:** Evelyn Vázquez, Preeti Juturu, Michelle Burroughs, Juliet McMullin, Ann M. Cheney

**Affiliations:** 1 Department of Social Medicine, Population and Public Health, School of Medicine, University of California, 900 University Avenue, Riverside, CA 92521-9800 USA; 2 Department of Community Health Sciences, UCLA Fielding School of Public Health, Los Angeles, CA USA; 3 Center for Healthy Communities, University of California, Riverside, USA; 4 Department of Family Medicine, School of Medicine, University of California, Irvine, USA

**Keywords:** Psychological trauma, Communities of color, Mistrust, Health disparities

## Abstract

Historical, cultural, and social trauma, along with social determinants of health (SDOH), shape health outcomes, attitudes toward medicine, government, and health behaviors among communities of color in the United States (U.S.). This study explores how trauma and fear influence COVID-19 testing and vaccination among Black/African American, Latinx/Indigenous Latin American, and Native American/Indigenous communities. Leveraging community-based participatory research methods, we conducted 11 virtual focus groups from January to March of 2021 with Black/African American (*n* = 4), Latinx/Indigenous Latin American (*n* = 4), and Native American/Indigenous (*n* = 3) identifying community members in Inland Southern California. Our team employed rapid analytic approaches (e.g., template and matrix analysis) to summarize data and identify themes across focus groups and used theories of intersectionality and trauma to meaningfully interpret study findings. Historical, cultural, and social trauma induce fear and mistrust in public health and medical institutions influencing COVID-19 testing and vaccination decisions in communities of color in Inland Southern California. This work showcases the need for culturally and structurally sensitive community-based health interventions that attend to the historical, cultural, and social traumas unique to racial/ethnic minority populations in the U.S. that underlie fear and mistrust of medical, scientific, and governmental institutions.

## Introduction

Communities of color in the United States (U.S.) have historically been affected by social determinants of health (SDOH), including, but not limited to: income, immigration status, and geographical location (Coroiu et al., 2020). SDOH are the intersecting conditions that influence health outcomes. Furthermore, SDOH adversely shape the life course and quality of life (QoL) for communities of color and perpetuate health inequities (Selden & Terceira, 2020; Tan et al., 2022). The multidimensional nature of SDOH and its power across multiple spheres of influence (e.g., intra and inter-personal, family, community, society) have exacerbated health inequities and racial health disparities in the current COVID-19 pandemic (Dalsania et al., 2022). This is evident in the disproportionate burden among racial and ethnic minority populations’ experiences of adverse COVID-19-related outcomes, such as high levels of hospitalization, morbidity, and mortality (Centers for Disease Control and Prevention 2021).

The COVID-19 pandemic has revealed existing “fault lines” (i.e., inequities; Snowden, 2019) of society and barriers to accessing culturally and structurally competent public health information, testing, and vaccination for marginalized communities of color (Carson et al., 2021; Gehlbach et al., 2022; Vázquez et al., 2021). While there is greater empirical understanding of the SDOH and their contribution to COVID-19-related health inequities, there is limited explanation of trauma’s role in influencing fear, hesitancy, and mistrust in public health and medical institutions among communities of color amidst the COVID-19 pandemic.

We explore how SDOH and structural determinants of health (e.g., structural racism) affect COVID-19 decision-making among Black/African American, Latinx/Indigenous Latin American, and Native American/Indigenous communities in Inland Southern California during the pandemic. We report findings from 11 focus groups and discuss the need for community-based interventions to address SDOH and their effects on collective grief with the potential to facilitate community healing and promote emotional wellbeing. Furthermore, based on our analysis of participants’ narratives across focus groups, we developed a conceptual model, which we refer to as the “Continuum of Trauma,” to illustrate trauma and its effects as it crosses temporal, cultural, and social spaces among communities of color. Consistent with our findings, we argue that collective experiences of trauma across this continuum have induced fear, hesitancy, and institutional mistrust (e.g., lack of trust in healthcare systems, public health authorities, and government departments) among marginalized communities. The proposed Continuum of Trauma model describes how and why multiple forms of trauma inform COVID-19-related health decisions such as testing and vaccination among communities of color.

## A “Continuum of Trauma” and its Effects in Communities of Color

Health inequity scholars have described three main types of traumas that affect communities of color: historical trauma, cultural trauma, and social trauma. These traumas may co-exist and can affect diverse social structures and institutions, including “cultural memory, language, self-rule, place, family system, economic resources, and healing systems” (Wiechelt et al., 2020: p. 175).

Historical trauma is described as “the cumulative and collective psychological and emotional injury sustained over a lifetime and across generations resulting from massive group trauma experiences” (Sotero, 2009: p. 96). Communities historically subjected to long-term massive group trauma experience the “cumulative and psychological wounding, over the lifespan and across generations” (Yellow Horse Brave Heart 1998: p. 7), known as intergenerational trauma. Historical trauma permeates all aspects of communities, such as their belief systems, coping strategies, the culture of emotions, and trust/mistrust towards dominant perspectives or institutions. Historical trauma is a predecessor of racial trauma theory (Chioneso et al., 2020), and it reflects the effects of societal oppression for historically marginalized racial and ethnic minorities. We conceptualize historical trauma as the result of unresolved historical grief.

Cultural trauma is defined as “an overwhelming and often ongoing physical or psychological assault or stressor perpetuated by an oppressive dominant group on the culture of a group of people sharing a specific group-based identity/affiliation (e.g., race/ethnicity, nationality, religion)” (Subica & Link, 2022). According to Wiechelt, et al. (2020), “[r]adical changes in the technological, economic, or political conditions (e.g., revolution, market collapse, forced immigration or deportation, genocide, terrorism, violence, the assassination of a political leader) can affect core values, beliefs, and norms, creating cultural disorientation and possible cultural trauma” (p. 172–173). Due to cultural trauma, communities of color can experience and suffer from an “erosion of their shared identity and the loss of familiar social structures” (Wiechelt et al., 2020: p. 175). We conceptualize cultural trauma as the result of constant assaults on the collective identity by hegemonic or dominant groups.

Social trauma has been described from a clinical and socio-psychological perspective (Hamburger, 2020). From a clinical perspective, this form of trauma entails the posttraumatic disorders at the group level caused by societal violence, abuse, or genocide. From a socio-psychological approach, social trauma is centered around long-term social interaction and processes (Bjornsson et al., 2020), such as rejection, neglect, and humiliation, that may represent a social threat to the family, the group, or the inter-group level. We conceptualize social trauma as the result of long-term social abuses and a threat to sense of belonging.

### Trauma in Black/African American Communities

Black/African American communities have endured immeasurable traumatic events related to race on a national and global scale, e.g., the institution of chattel slavery, the Rosewood massacre of 1923, the government-sponsored Tuskegee Syphilis Experiment from 1932 to 1972, the Los Angeles riots of 1992 and, more recently, overrepresentation in the school-to-prison pipeline and incidents of police brutality (Howard, 2016; Klein & Lopez, 2022; Lynch, 2016). Generations later, African Americans continue to carry their history’s mental and social scars, including feelings of inferiority, powerlessness, and problems with self-identity (Carter, 2007). Furthermore, scholars have explained the danger of systems of oppression and massive violence and the deleterious effects of racial trauma, an ongoing consequence of historical trauma, on the mental health and well-being of Black/African American communities across surviving generations (Chioneso et al., 2020).

### Trauma in Latinx/Indigenous Latin American Communities

Trauma among Latinx and Indigenous Latin American immigrant communities in the U.S. is associated with a perceived low sense of wellbeing and a high prevalence of mental health conditions, including depressive symptoms, substance use and addiction, post-traumatic stress disorder (PTSD), and diminished life satisfaction (Garcini et al., 2022). The nature of trauma experienced by Latinx immigrants is associated with bereavement and has been described as a loss of roots, including physical geography, lack of people who can be trusted or who care about them, as well as shifts in belief systems, home country social status, and sense of community (Hill et al., 2021). Latinx communities have also suffered from unethical actions of investigators and the U.S. government in nonconsensual human medical experiments such as the 1946 to 1948 Guatemalan Sexually transmitted disease experiments (Rodriguez & Garcia, 2013).

Scholars have explained that the ethno-racial trauma among Latinx immigrant communities living in the U.S. is grounded in a legacy of interlocking systems of oppression, including racism, sexism, and anti-immigrant laws, policies, and practices (Chavez-Duenas et al., 2019). Fear of deportation diminishes the perceived sense of psychological safety, security, and trust (Rojas-Flores et al., 2017). Ethno-racial trauma results from witnessing or experiencing discrimination, violence, intimidation, and threats of harm directed at ethno-racial minority groups. In this context, undocumented immigrants experience multiple vulnerabilities and layers of inequities, including susceptibility to abuse, exploitation, and deportation (Adames & Chavez-Dueñas, 2017) increasing the risk of exposure to ethno-racial trauma and migratory grief (Achotequi, 2000; Falzarano et al., 2022).

### Trauma in Native American/Indigenous Communities

Native American/Indigenous communities in the U.S. have experienced multiple forms of trauma with the beginnings of settler colonialism to the present. Such trauma represents structural violence, a violence that is often invisible and plays out in seemingly ordinary ways, which continues through refusal of the government to honor treaty obligations, the taking and destruction of land (i.e., pipeline projects), the taking of children by social workers who are incentivized to remove Native children from their families (Sullivan & Walters, 2011), recent legal threats to the Indian Child Welfare Act (ICWA), and the continued erasure of data on the high rates of missing and murdered Indigenous people. As Kirmayer et al. (2014) emphasize, historical trauma is connected to both past and ongoing oppression. Early conceptualizations of historical trauma by Yellow Horse Brave Heart (2003) and by Duran and Duran (1995) as a “soul wound” focus on the relational aspects of Indigenous knowledge and practice affected by systematic oppression, physical, and socio-cultural genocide by settler-colonialists, the loss of physical/natural environments that sustained families and communities, the boarding school era, and forced relocation to reservations or removal from ancestral lands altogether.

Similar to soul wounds, Indigenous historical trauma (IHT) emphasizes the violence of colonization as collective trauma, ancestral adversity, intergenerational transmission, and compromising the well-being and health of descendent generations (Gone et al., 2019). The Tribal Nations in the occupying California counties of Riverside and San Bernardino in Inland Southern California have experienced many of the abuses—massacre, removal and forced relocation to the reservations, boarding schools which exist to this day, disruption of economic system, and ability to gather food (Milanovich, 2021). All present persistent actions described as leading to historical trauma. Gone et al., (2019) also make a clear distinction that historical trauma is more readily attributed to Native Americans/Indigenous groups than racialized trauma.

A key distinction is that the structural violence affecting Indigenous Native American tribes is not racial identity but a political tribal identity. The oppression is enacted against tribal nations and their citizens who have signed international treaties with the U.S. Indeed, the current Supreme Court Hearings on ICWA hinge on an understanding that Indigenous Native Americans are defending political rights and not fighting racial discrimination against colonizers. To this day, Native Americans/Indigenous persons must protect their land, families, rights to health care, and lives from a government with whom they have signed treaties.

## Project Overview

This work was part of the larger state-wide effort, Share Trust Organize Partner COVID-19 California Alliance (STOP COVID-19 CA) funded by the National Institutes of Health Community Engagement Alliance (CEAL), to increase equity in COVID-19 testing, vaccination, and clinical trial participation in vulnerable and underserved communities throughout California (https://stopcovid-19ca.org/). This manuscript presents findings from research focused on equity in COVID-19 testing and vaccination conducted at one of the 11 sites in the STOP COVID-19 CA CEAL network. This project explored the lived experiences of communities of color living in Inland Southern California. Before the start of data collection, we obtained ethical approval for the study from the Institutional Review Board at the University of California, Riverside.

We employed community-based participatory research (CBPR), an approach to research addressing the concerns and needs salient to marginalized communities (Zimmerman, 2021). CBPR advocates for equitable distribution of power, shared learning, knowledge co-creation, community-based decision-making, and culturally sensitive resource sharing among community and academic partners (Minkler & Wallerstein, 2008; van de Sande & Schwartz, 2017). We applied CBPR principles via convening three engagement teams led by an academic and community investigator partnership to engage Black/African American, Latinx/Indigenous Latin American, and Native American/Indigenous communities. Each team included representation from the community and members of their respective community were engaged throughout the project. In line with CBPR principles, we conveyed a community advisory board (CAB) with diverse stakeholders, including community members, students, community researchers, healthcare and public health representatives, and academics. The CAB guided the research and engagement of community in each aspect of the project.

## Study Setting

This study focuses on the structural and social factors contributing to COVID-19-related testing and vaccine decisions in underserved and marginalized communities in Inland Southern California, a region known for its racial and ethnic diversity. Inland Southern California includes both Riverside and San Bernardino Counties covering nearly 30,000 square miles with a population of more than 4 million. This population is greater than 30 US states and is the 13^th^ largest metropolitan area in the US. The area is the ancestral homelands of the several Native American/Indigenous tribes, the Cahuilla, Tongva, Luiseño, and Serrano peoples (UCR Native American Student Programs n.d.). There are 12 federally recognized tribes in Riverside and San Bernardino Counties, the second largest number of tribal governments located in a single region in the U.S. The locations of tribal lands span a 400-mile radius, which includes both urban and rural areas. It is also home to a large Latino population with ties to Mexico. In this region, Latinos, primarily of Mexican origin with a smaller number of Puerto Ricans, Salvadorans, Guatemalans, and Indigenous Amerindian groups, are the “minority” majority population with over 50% of the population identifying as Latino/Hispanic. Blacks/African Americans also have a significant presence in the region with a history dating back to the Spanish colonization and increasing during the Great Migration of the 20^th^ century. Most recently, prohibitive housing costs have pushed many Blacks/African Americans similar to other racial/ethnic groups out of urban centers such as Los Angeles to the inland region in search of more affordable housing.

Racial-ethnic groups in this region have continuous histories of discrimination, oppression, and prejudice that profoundly contribute to health disparities. For instance, according to the Center for Disease Control/Agency for Toxic Substances and Disease Registry (CDC/ATSDR), both Riverside and San Bernardino Counties rank high on the social vulnerability index, which harms health and well-being of communities living in the region. Exposure to structural and social inequities deter many from accessing healthcare services for COVID-19 placing these racial/ethnic groups at increased risk for COVID-19 morbidity and mortality (Gehlbach et al., 2022).

## Participant Eligibility and Recruitment

Participants were eligible if they met the following criteria: (1) 18 years of age or older, (2) self-identified as Black/African American, Latinx, Hispanic or Indigenous Latin American, and/or Native American/Indigenous, (3) spoke English, Spanish, and/or Purépecha, and (4) lived in Inland Southern California (i.e., Riverside and San Bernardino Counties). Team members recruited community members into focus groups by distributing study flyers and study contact information through relevant social, cultural, institutional, and professional networks. In addition, participants were recruited from various channels including social media, word of mouth, and existing listservs.

## Qualitative Data Collection

From January to March 2021, we conducted 11 virtual focus groups of 5 to 10 people, each with community members self-identifying as Black/African American (*n* = 4), Latinx/Indigenous Latin American (*n* = 4), and/or Native American/Indigenous (*n* = 3) to elicit shared information on the cultural and structural factors shaping COVID-19 testing and vaccination in marginalized and vulnerable communities in Inland Southern California. A total of 89 participants attended a focus group with 42% identifying as Latinx or Indigenous Latin American, 36% as Black/African American, and 22.0% as Native American/Indigenous. Most (57.9%) self-identified as female, 39.5% as male, and 2.6% as other. Nearly two-thirds (74.4%) had been tested for COVID-19 of whom 39.1% tested positive. Over a two-thirds (65.1%) had either gotten the COVID-19 vaccine or planned to get it (see Cheney et al. 2021 for additional demographic characteristics).

Prior to data collection, we developed a qualitative research training to build the capacity of diverse community members and university students to partner in research. A total of 52 people (20 students, 26 community members, 6 community-academic team members) participated in a four-part qualitative training series on qualitative research, ethics in research, data collection, analysis, and interpretation. Community-academic team members led the trainings in English and Spanish with 65.4% attending in English and 34.6% in Spanish. Of the 52 trainees, 6 to 8 community members or students per engagement team opted to facilitate focus groups and/or serve as notetakers and continue with data analysis and interpretation.

A semi-structured interview guide with open-ended questions was used during the focus groups. Questions sought to elicit the socio-cultural and structural factors influencing decisions around COVID-19 testing and vaccination in the three identified communities. The interview began with open-ended questions on community members’ understanding of the virus, its spread, and COVID-19 testing and vaccination, as well as collective understandings of trauma and inequity in the COVID-19 pandemic. Each engagement team conducted focus groups via Zoom video conferencing, which were audio recorded and professionally transcribed. In addition, participants completed a socio-demographic survey either via a link to a Qualtrics survey (self-administered) or by phone (a team member administered the survey as an interview).

## Data Analysis

Our analysis focused on trauma and how it manifested in the COVID-19 pandemic across the diverse groups engaged in our research. We draw on the theories of intersectionality (Crenshaw, 1991, 1994) and trauma to illustrate how the intersection of race and gender with multiple marginalized identities, such as being a person of color, a low-income patient, undocumented, and a non-English speaker, shape experiences of historical, cultural, and social trauma across diverse communities of color and inform healthcare decision making in the COVID-19 pandemic.

Transcripts from the 11 focus groups were transcribed and analyzed using template and matrix analysis, a rapid analytic approach that facilitates the summarization of textual data and theme identification and constant comparison (Averill, 2002; Nadin & Cassell, 2004). We used a blended deductive (i.e., existing categories based on the interview guide) and inductive (identification of emergent themes) and iterative process involving consensus building among analytic team members. Because this approach does not involve code application and assessment of interrater reliability, we ensured rigor in data analysis through interactive group discussions and consensus building during data synthesis and interpretation. Each engagement team analyzed the qualitative data from their respective focus groups. All analytic team members had either participated in the training series or was a member of the community-academic partnership.

As a first step, a summary template that included the key interview domains that mirrored the interview guide was created. Key interview domains included: (1) attitudes and beliefs about the coronavirus, its spread, and public health measures, (2) COVID-19 testing and vaccination access, and (3) trauma and inequality in the COVID-19 pandemic. Next, members of each engagement team read, line by line, the transcripts from their respective focus groups along with the meeting notes taken during the focus groups, and inserted data, including illustrative participant quotes, in the templates. Then, each team met to discuss the data in the templates, condense it, and insert the data from the templates into a single matrix to organize the responses summarized in the templates. Therefore, the matrix included condensed text from the templates (as rows) by key interviews domains (as columns), which facilitated pattern and theme identification across focus groups by community. The final matrix from each team’s analysis was then input into a matrix that included patterns and themes from across three matrices completed by each engagement team. This final matrix included patterns and themes from each engagement team’s matrix (as rows) by key interview domains (as columns) and facilitated a comparative analysis across cases (i.e., Black/African American, Latinx/Indigenous Latin American, Native American/Indigenous) and interpretation of key themes across the three communities.

## Findings

**Fig. 1:**
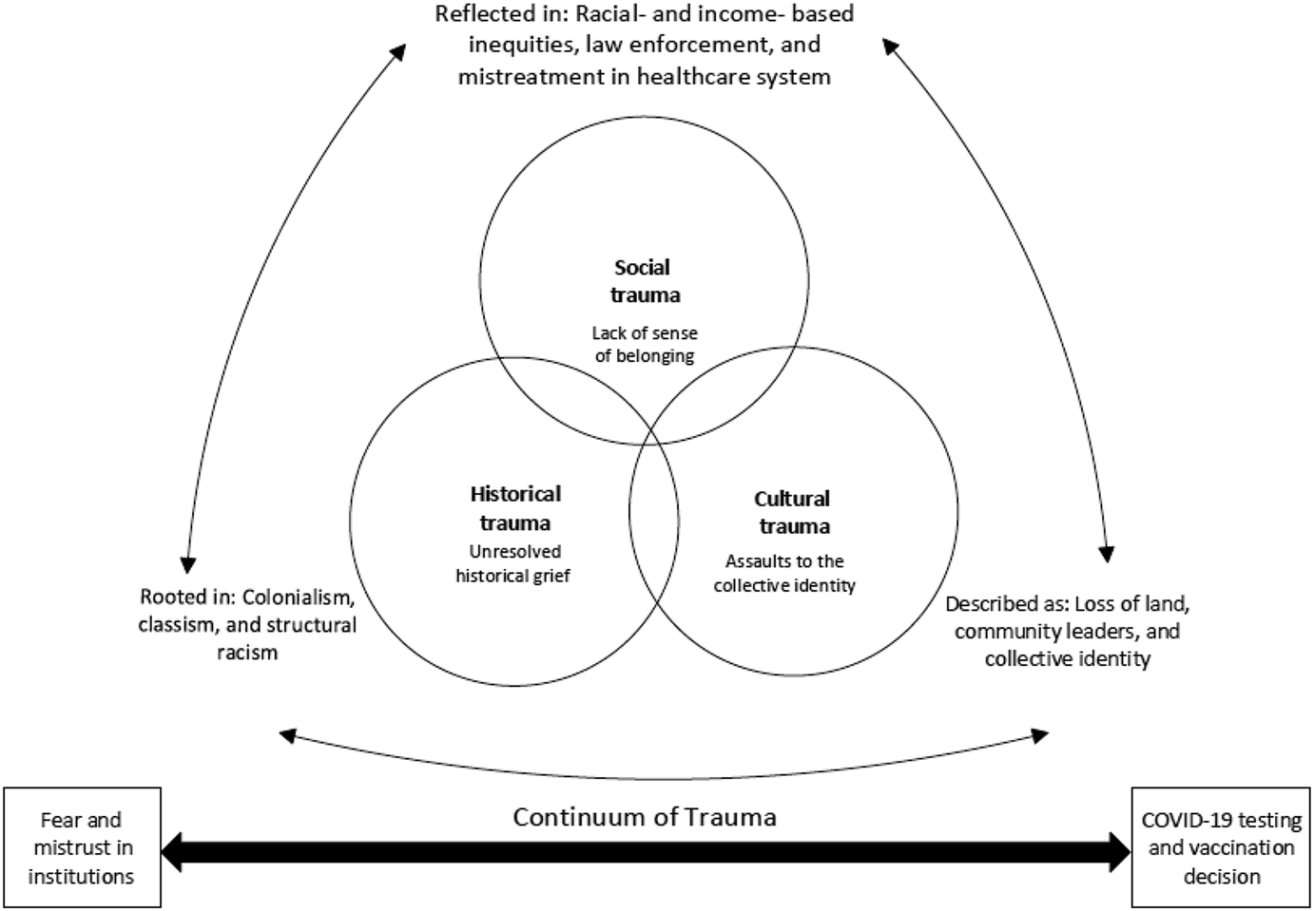
Continuum of Trauma among communities of color during the COVID-19 pandemic

In line with inductive approaches to textual data, from our comparative analysis we developed the “Continuum of Trauma” model. As seen in Figure 1, the cross-case data analysis elicited and indicated that various levels of trauma, including historically based trauma (stemming from colonialism, classism, and structural racism), cultural trauma (loss of land, community leaders such as priests and elders, and collective identity), and social trauma (racial- and income-based inequities, law enforcement, and mistreatment in healthcare systems) contribute to collective trauma, fear, and mistrust toward COVID-19 testing and vaccination.

These traumas are interconnected and interrelated and give meaning to the attitudes and belief systems that induce fear and mistrust in institutions and shape healthcare decision-making around COVID-19 testing and vaccination. Below, we use participants’ quotes from the focus groups to illustrate the manifestation of trauma along the continuum among communities of color during the COVID-19 pandemic.

The dominant Eurocentric culture in the U.S., is characterized by hierarchical, asymmetrical, and exclusionary interactions stemming from a history of colonialism and social divisions by class, education, income, race/ethnicity, indigeneity, immigration status, and English proficiency, which deeply affect communities of color. In addition, the medical hegemony of Eurocentric paradigms influences discrimination against marginalized racial, ethnic, and political communities. This culture promotes values and norms that reproduce privilege (in terms of power, services, and resource access) for middle-class, white, English-speaking, documented communities while reinforcing—intentionally and unintentionally—the oppression, marginalization, and exclusion of communities of color from healthcare systems, contributing to COVID-19-related health disparities.

### Historical Trauma: Unresolved Historical Grief

Across focus groups, participants discussed historically based trauma as grounded in histories of government and public health abuses. Comments such as communities of color being used as “guinea pigs” for medical treatments, being “lied to” and “taken advantage of” by the government were common experiences among participants. As a Native American/Indigenous community member commented: “I don’t trust doctors. I don’t trust the government. I don’t trust anything.”

Across the focus groups with Black/African American and Native American/Indigenous community members, participants talked about the falsehoods and lies enacted by the U.S. government:

Those of us who are old enough to remember the syphilis Tuskegee, the lie in just all of those situations that happened to black people being experimented on, even lobotomies, castration all of that, that weighs heavy for all of us. (African American community member)

[W]ith the measles disease blankets or chicken pox, smallpox, or whatever they were infested with that they gave us, and the hysterectomies without permission from the women that were given, the government. That’s what we’re calling them. But anyway, [the government] shouldn’t be trusted. (Native American community member)

Discussions about the Tuskegee Syphilis study were salient in focus groups with Black/African American community members. A participant commented:

. . . it’s very valid that the history we have with the government, especially in cases of medical institutions, that Tuskegee is not the only thing. There’s a long history of abuse and trauma when it comes to our relationship with the medical institutions in this country.

Whereas across the focus groups with Latinx/Indigenous Latin American community members, classism, another form of historically based trauma, was pronounced:

In my community, they say that the powerful [the rich people] have more attention [in the hospitals], and the humble [low-income people] is the one who has the least attention. Those people who have money are very well cared for and are the ones who they pay more attention to, and people who really do not have economic resources, they see them as [they are] nothing. Like they feel that... they don’t give them [the] care they need. They [people in the hospitals] do not provide the same care as to a person who arrives and... has money.

Issues of classism were prevalent among all communities included in our study.

### Cultural Trauma: Assaults on the Collective Identity

Participants shared how the COVID-19 pandemic affected their collective identity, particularly in disruptions to their relationality and loss of ancestral knowledge and continuation of social life and values due to the passing of key figures (e.g., elders or priests). A Native American/Indigenous community member shared the following:

I think that it [the COVID-19 pandemic] changes our tradition and our outlook on things, because we need that closure. We need to see that person to say goodbye before we can close that box. And we don’t have that no more. And it's really, really hard. And that’s just part of our tradition, and somehow, we have to try to move around it. It’s hard enough moving around it when you do close that box, but when you don’t, not being able to say your goodbyes or whatever, that makes it even more tragic I think for us as Indian people.

The passing of key figures such as elders or priests altered collective coping strategies, leading to feelings of desolation. Another member of the Native American/Indigenous community similarly shared:

I feel like I’m sinking below the level of sadness, because I can’t try to rise above it because there’s no closure to nothing. And we’ve been doing a lot. And we’ve lost family members too to the COVID, and we have some in the hospital right now fighting it [COVID-19].

Participants in the Latinx/Indigenous Latin American focus groups also discussed the loss of leaders in their community during the COVID-19 pandemic. As one participant said, “COVID took the priest.” Latinx participants talked about the loss of two priest because of COVID in less than one year and how that affected the morale of their communities.

### Social Trauma: Racial and Income-Based Marginalization

The characteristics of social trauma and racial- and income-based inequities, including unstable employment, overcrowded housing, and mistreatment in healthcare settings, were pronounced in the current pandemic. The insufficient financial resources and economic opportunity in communities of color was noticeable in the early months of the pandemic. “There are no such resources and... well, we’re going to die. I feel like that’s what a lot of people think. Because I live [paycheck to paycheck],” shared a Black/African American participant. Contingent, low-wage or essential labor pushed many caregivers to work outside the home in the early days of the pandemic leaving children behind. A participant in the Black/African American focus group commented:

Parents aren’t able to [not] go to work… and leave the kids at home. It doesn’t create a very good environment for kids to come up in. . . it’s not very secure. It can cause a lot of stress on people.

In addition to job-related stress and childcare concerns, many faced housing inequities. Because of limited financial resources, many families of color live in intergenerational or multifamily households in small and overcrowded homes—environments germane to virus spread. “I think that’s why we see so many [health] disparities within families having to live in such large numbers in such small areas,” explained a Black/African American participant. Not only did limited finances affect housing, but it also informed medical and healthcare decisions. A participant in the Latinx/Indigenous Latin American focus group commented:

I don’t have much money. I can’t get healthcare. . . . Many people still do not use healthcare services because they consider: ‘I’ll pay my rent, or I’ll pay for healthcare.’ Because they are more limited to being able to have [financial] resources to care for themselves or not have resources at all.

As participants shared, communities of color experience discrimination in healthcare settings due to limited financial and economic resources. This discrimination is a powerful factor facilitating better treatment in high-income neighborhoods compared to low-income communities and instills fear and mistrust in medicine and healthcare services. The following comment shared in a focus group with Latinx/Indigenous Latin American community members illustrates this point:

Sometimes they look at you like you have fleas, or I don’t know what they think. The people in the hospitals [healthcare professionals] sometimes ignore you [because you speak Spanish] . . . and it’s not right . . . I’m not going to complain because if I go the second time, or the third time [to the same hospital] they will no longer want to attend me or they will recognize me . . . [and treat me badly].

Fear of being singled out and the potential for mistreatment silences voices within healthcare settings.

## Fear and Mistrust in Institutions

As seen in Figure 1, the continuum of trauma exemplifies how the intersection of historical, cultural, and social trauma has contributed to fear and mistrust in institutions—government, medicine, and public health. These different yet harmful levels of trauma intersect and shape decision making around COVID-19 testing and vaccination in the current pandemic. The pandemic is “unpredictable” and has “interrupted” daily life, bringing to light internalized notions of unresolved historical grief, assaults on collective identity, and marginalization.

Such histories of trauma enacted by public institutions (e.g., the U.S. government) inform behaviors toward COVID-19 testing and vaccination. A participant in a Native American/Indigenous focus group shared:

We’ve been lied to so many times, our communities. We’ve been taken advantage of. We’ve been guinea pigs. I do not trust them [the U.S. government] for that reason. … I’m really hesitant to do it [the COVID-19 vaccine] . . . I think the U.S. [government] could’ve had something to do with it in the very beginning. I don’t trust the U.S. as you’re seeing why because of all the traumatization from before and we’ve been lied to, we’ve been taken advantage of. I don’t want to see it again.

Fear and mistrust of institutions, specifically the government and public health, was echoed throughout the focus groups. A participant in the Black/African American focus group shared:

That is the fear [among Black/African American communities]. That is the mental health and the stress that goes along with this pandemic and our decision even to make the choice of if we’re going to take the [COVID-19] vaccine or not. I think that a lot that is going on here is driven by fear. I think that is what needs to be dealt with.

Participants in the Latinx/Indigenous Latin American focus groups talked about fear and mistrust in public health, as well as fear of job loss as undermining COVID-19 related healthcare decision making:

People have [fear], because there was no [COVID-19 testing] at the beginning [of the pandemic]. They [public health testing site] do it in the morning from 8 am to 12 pm. So, people would say: ‘Why would I lose my job to go to get tested?’ That's why not many people went to get tested. But I think they started doing it in the afternoon, like in the church, when the priest was still there. But COVID took the priest. So now, we don’t know where to go to get tested.

The above quotes exemplify how collective experiences across the Continuum of Trauma contribute to health inequities in the COVID-19 pandemic. A participant in a focus group with members of the Black/African American community explained well the underlying causes of such trauma and disparity:

I think a lot of things can be done about the racism in this country and the institutions. I don’t think any problem can be solved until that has been taken care of. I think that when we’re talking about health disparities, we’re talking about lack of education, access to resources. I don’t think… so we can get people to take the vaccine, but the trauma will always still be there until the issue of racism is solved . . .

## Discussion

The current pandemic is one of multiple epidemics—COVID-19, racism, fear, and mistrust of institutions. Furthermore, the COVID-19 pandemic has accelerated syndemic traumatic stress, emotional suffering, and health inequities across populations (Azevedo et al., 2022). As our study shows, trauma exists along a continuum and involves historical, social, and cultural traumas that spans the life course and cross generations contributing to fear and mistrust in institutions and health disparities in the COVID-19 pandemic (Priest & Williams, 2021). The Continuum of Trauma gives perspective to why many communities of color distrust government institutions, are fearful of seeking medical care, and are reluctant to get tested and vaccinated for COVID-19. The findings speak to the medical hegemony of western Eurocentric paradigms that (re)produce classist and racist policies and practices within healthcare and public health institutions that work to discriminate against marginalized racial, ethnic, and political communities (Baer et al., 2013; White & Chanoff, 2011). They also speak to how the culture within such institutions promote values and norms that reproduce privilege (in terms of power, services, and resource access) for middle-class, white, English-speaking, documented communities. While reinforcing—intentionally and unintentionally—the oppression, marginalization, and exclusion of communities of color from healthcare and public health systems, contributing to COVID-19 related health disparities and the COVID-19 global mental health syndemic (Azevedo et al., 2022).

As the lived realities of participants in our study underscore, it is within these spaces (healthcare and public health) that communities of color, directly and indirectly, experience, and/or witness various forms of trauma (historical, cultural, social) due to real or misinterpreted racism (Smith., 2010). This trauma contributes to beliefs that the COVID-19 vaccination may be more detrimental to health than protective of health, contributing to health disparities amidst the COVID-19 pandemic (John-Henderson & Ginty, 2020).

Participants’ narratives across racial/ethnic communities show how the dominant Eurocentric culture in the U.S., characterized by hierarchical, asymmetrical, and exclusionary interactions, stems from a history of colonialism and social divisions by class, education, income, race/ethnicity, indigeneity, immigration status, and English proficiency. It is along these “fault lines” created by social and structural inequities that epidemics and pandemics flows (Snowden, 2019). This is exactly what we see among communities of color in Inland Southern California in the COVID-19 pandemic—they are the most vulnerable to the virus and its spread due to underlying disease burden (asthma, obesity), living and working conditions, and fear and mistrust. As others point out, the pandemic has highlighted how structural racism reproduces racialized “otherness” and structures and oppressive systems within society that produce and perpetuate health disparities (Priest & Williams, 2021). Racism underlies fear and mistrust in institutions, specifically medicine, science, and public health, shaping COVID-19-related testing and vaccine decision-making among historically disadvantaged populations, including low income communities of color (Gehlbach et al., 2022; Vázquez et al., 2021). Mistrust in medicine and science has also shaped the discourse around racial and ethnic disparities in COVID-19 vaccine uptake, shaping vaccination decision making among African American, Latinx, Indigenous, and Asian American populations within the U.S. (Corbie-Smith, 2021).

Since the start of the pandemic, structural racism, which we conceptualize as spanning the Continuum of Trauma and being embedded within institutions and the norms of social life, has been at the core of COVID-19 related health disparities. Structural racism has obstructed access to vital health care services and resources, including credible/culturally inclusive public health information for racial and ethnic minorities and accessible locations to receive COVID-19 vaccines (Boyd, 2021). As shown in our study, structural racism surfaced in health policymaking and practices (e.g., the when, where, and for whom of COVID-19 testing sites), discourses of COVID-19 healthcare (e.g., higher quality care for English-speaking patients), as well as access to societal resources (e.g., income and economic stability), and opportunity structures (e.g., ability to work from home) (Holden et al., 2022; Johnson, 2020; Jones, 2000). Structural racism, as it transverses the Continuum of Trauma, has perpetuated fear and mistrust of institutions among communities of color, forcing many to critically consider the purpose of testing and vaccination (e.g., government schemes to harm racial/ethnic minority populations) and dissuading some from accessing COVID-19 testing and vaccination services.

## Public Health Implications

**Fig. 2:**
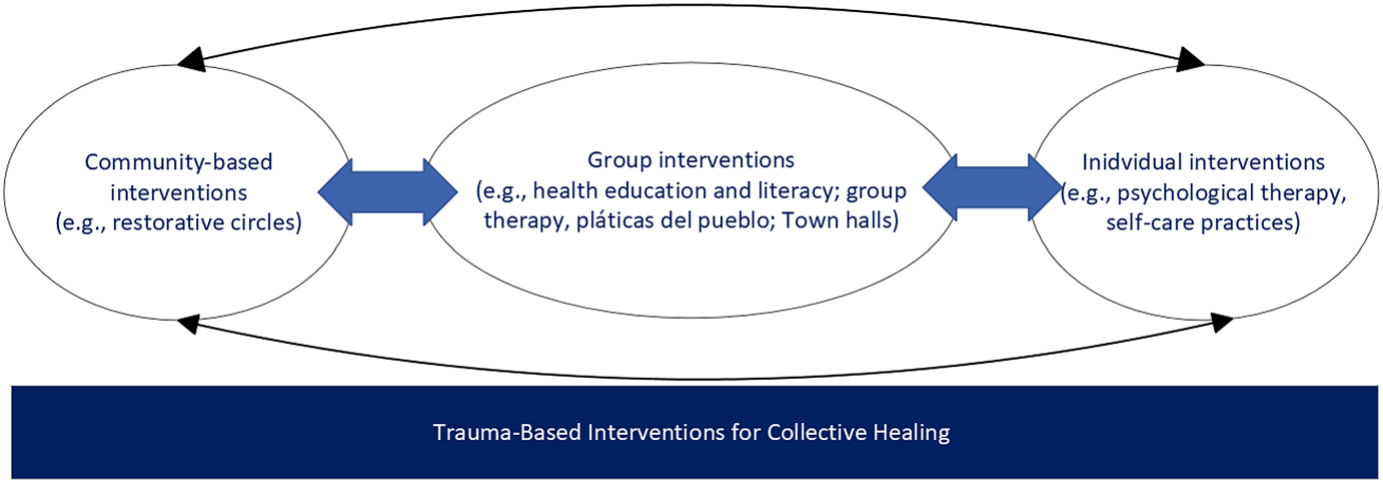
Trauma-based Interventions for Collective Healing

Our findings indicate the need for community-based trauma approaches for collective healing. Transformative interventions, known as facilitators of collective liberation and wellbeing, are needed to address the fear and mistrust of institutions (Riemer et al., 2020). These interventions aim to change the social order by transforming three types of power: political, economic, and social. Transformative interventions are informed by culturally sensitive and structurally responsive approaches that help to increase a perceived sense of mattering, empowerment, and further liberation in communities of color. These interventions can promote community-level health equity and freedom from trauma by promoting health education and literacy, prevention and health promotion, and access to community-based interventions developed by persons/communities of color for communities of color (see Fig. 2).

Restorative circles, an example of transformative anti-racist interventions (Adkins-Jackson et al., 2023) that promote culturally and contextually sensitive community-based interventions (Vázquez et al. Forthcoming) help to mitigate psychological trauma and mental health conditions among communities of color. These circles are informed by community psychology approaches (such as social justice) and psychologies of liberation. The goal of restorative circles is to create safe spaces for collective healing. During the circles, attendees can share their lived experiences, traumas, and challenges without the fear of being rejected, stigmatized, or judged. These circles create a collective space for grief and healing, foster trust in healthcare systems, address health literacy concerns (e.g., available resources are free and in Spanish), and increase support, social connectedness, and sense of belonging.

COVID-19 related health disparities stem from historically rooted injustices (Dalsania et al., 2022), such as medical experimentation on communities of color, which have, over centuries, contributed to mistrust and suspicion of institutions associated with authority and the production of medical knowledge, including the academic/scientific community, public health and healthcare systems, and government. Our findings reinforce the need to respond to the various forms of trauma (historical, cultural, social) and their effects on psychological wellbeing and health equity among communities of color by developing, in collaboration with communities of color, culturally and structurally sensitive community-based interventions that will help to heal historically marginalized and vulnerable communities as well as create a just and safe healthcare service for all.

## Limitations and Next Steps

Study findings offer important insight into the socio-cultural and structural factors shaping COVID-19 testing and vaccination among communities of color in Inland Southern California. However, several limitations should be considered when interpreting the findings. Because of the qualitative nature of our project, the findings do not pretend to demonstrate any generalizability and rather provide a description and shared experience of how trauma informs decision-making related to COVID-19 testing and vaccination among communities of color in Inland Southern California. That said, our findings may not represent the experiences or perspectives of communities of color in different geographic regions of the U.S. such as the south or Midwest as their unique historical and current-day experiences would shape trauma exposures. Additionally, while Figure 1 illustrates the interrelationships and interconnectedness of historical, cultural, and social trauma, the presentation of the results may undermine the goal to demonstrate their connectivity. This is a general limitation of presenting qualitative findings as if the themes/types of trauma are separate, when in fact, they are always informing each other.

Despite these limitations, this work informed subsequent steps for research focused on empowering communities to develop culturally and structurally relevant strategies to mitigate the effects of COVID-19 on their communities. This included working with community to use the findings to develop toolkits that addressed, in varied ways, the socio-cultural and structural inequities embedded within institutions. For instance, the Latinx/Indigenous Latin American engagement team designed and developed a mural and testimonial to address mistrust and fear in healthcare systems (Cheney et al., 2023). The Black/African American engagement team developed the STOP COVID-19 Black Community Resource Toolkit (Stop COVID-19: Black Community Workgroup 2022). The Native American/Indigenous engagement team developed an online COVID-19 toolkit with public service announcements, COVID-19 messages in indigenous languages, and a digital literacy guide during COVID-19 and beyond (Gathering of Good Minds (Chihuum Piiuywmk Inach) n.d.).

Furthermore, this Latinx/Indigenous Latin American engagement team has begun to address the trauma of collective grief and loss among vulnerable Latinx immigrant communities in the rural Inland Southern California through the implementation and evaluation of restorative circles. This work builds on the state-wide model and engages community health workers or promotoras de salud in addressing community mental health (Adkins-Jackson et al., 2023).

## Conclusion

Fear and mistrust in healthcare services and systems in the COVID-19 pandemic stems from historical trauma and colonizing practices in the history of medicine (e.g., forced sterilization, Tuskegee). It also reflects current social trauma linked to cruelty (e.g., criminalization, police brutality, excessive violence, and the militarization of the border) enacted by the U.S. government towards racial/ethnic minorities and cultural trauma such as loss of elders and knowledge. While we recognize that mistrust of institutions also exists among rural White populations in the US, we believe the factors that shape mistrust in medicine for such populations reflect upholding values of personal freedom and choice (Vázquez et al., 2022) rather than a response to exposure to the Continuum of Trauma. Experiences of trauma, especially historical and social trauma, deter communities of color from accessing health services more generally, which contributes to COVID-19 morbidity and mortality in the current pandemic exacerbating cultural trauma through the loss of esteemed elders and leaders, identity, and sense of community.

The Continuum of Trauma described in this article evidences the shared grief, assault on collective identity, and marginalization among racial and ethnic minorities in Inland Southern California. The long-term effects of historical trauma (systemic racism, classism, and colonialism) intersect with cultural and social trauma and exist today as a lack of care, respect, and nurturing of oppressed and marginalized communities. Racial and ethnic communities have been tremendously affected during the COVID-19 pandemic leading to new cycles of trauma, unresolved grief, and loss, and marginalization that reify fear and mistrust towards White Eurocentric culture that has actively sought to oppress and colonize the bodies, minds, and souls of communities of color. We argue that intersectional and transformative interventions that address COVID-19 related health disparities must be culturally and structurally responsive to address the intersecting trauma experiences of historically marginalized communities.
